# P-808. Nudging Strategies in Microbiology Laboratory Reports: An Updated Systematic Review

**DOI:** 10.1093/ofid/ofaf695.1016

**Published:** 2026-01-11

**Authors:** Esther Jeong, Bradley J Langford, Elizabeth Leung, Jenna Wong, Vaishnav Sivakumar, Glyneva Bradley-Ridout, Larissa Matukas

**Affiliations:** Unity Health Toronto, Toronto, ON, Canada; Public Health Ontario, Toronto, Ontario, Canada; Unity Health Toronto, University of Toronto, Toronto, Ontario, Canada; Unity Health Toronto, Toronto, ON, Canada; University of Waterloo, Waterloo, Ontario, Canada; University of Toronto, Toronto, Ontario, Canada; Unity Health, Toronto, Ontario, Canada

## Abstract

**Background:**

Nudging strategies in microbiology laboratory reports can influence decision-making by helping clinicians make more appropriate antimicrobial prescribing choices. We aimed to update a 2019 scoping review to evaluate the existing literature on nudging in microbiology laboratory reports and its association with antimicrobial prescribing.

**Figure 1:**
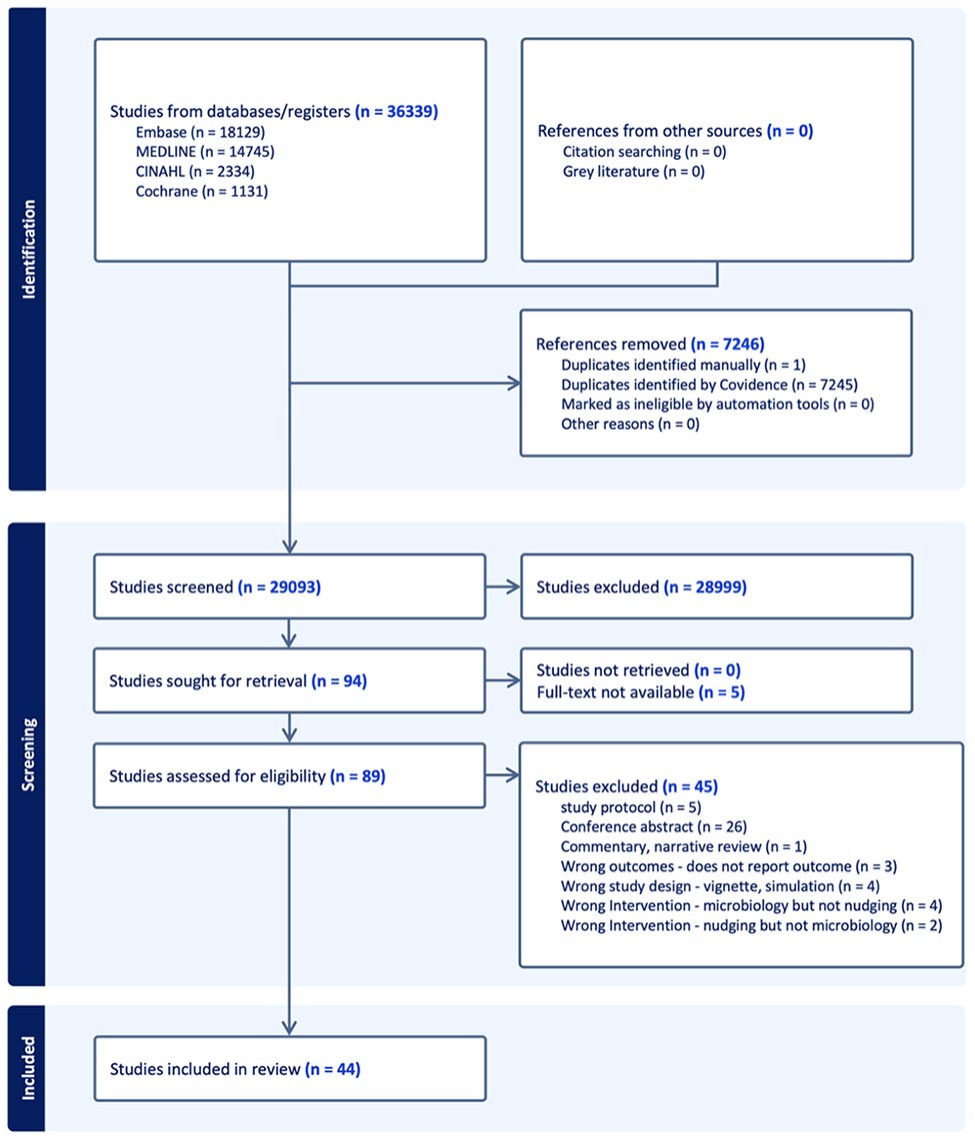
PRISMA Diagram

**Methods:**

In alignment with PRISMA reporting guidance, a comprehensive search was conducted across databases including Ovid MEDLINE, Embase, EBSCO CINAHL, Cochrane, and clinical trial registries, with no restrictions on language or start date. Key search terms targeted concepts of nudging, decision-making and behaviour, and microbiology. Eligible studies involved the use of nudging strategies in microbiology reports for all human populations in any healthcare setting, excluding non-interventional and simulated studies. The study protocol was registered a priori on PROSPERO (#CRD42024568670).

**Results:**

A total of 36,339 studies were identified. After title/abstract and full-text screening, 44 studies were included (Figure 1). The majority were conducted in inpatient settings (n=39, 89%), focusing on adult patient populations (n=43, 98%). Most studies were published in the past 5 years (n=32, 73%). Selective, restrictive, cascade antimicrobial reporting was the most common primary nudging strategy (n=34, 77%), followed by framing or comments/recommendations to provide interpretation of the results (n=10, 23%). Many studies (n=39, 89%) evaluated antibiotic use as an outcome, with nudging associated with reduced/improved prescribing in 29 (74%).

**Conclusion:**

Since our previous scoping review, the volume of research on nudging strategies in microbiology laboratory reports has grown substantially. The findings from this systematic review offer valuable insights for healthcare leaders and policy-makers, highlighting nudging practices that can be integrated into clinical microbiology workflows to promote responsible antimicrobial prescribing.

**Disclosures:**

All Authors: No reported disclosures

